# *AsNAC* Genes: Response to High Mercury Concentrations in *Allium sativum* Seed Clove

**DOI:** 10.3390/biotech14020027

**Published:** 2025-04-08

**Authors:** Brenda Mendoza-Almanza, María de la Luz Guerrero-González, Marcos Loredo-Tovias, María Elena García-Arreola, Catarina Loredo-Osti, Erika Padilla-Ortega, Pablo Delgado-Sánchez

**Affiliations:** 1 Biotechnology Laboratory, Faculty of Agronomy and Veterinary, Universidad Autónoma de San Luis Potosí, Soledad de Graciano Sánchez CP 78439, SLP., Mexico; a348509@alumnos.uaslp.mx (B.M.-A.); luz.guerrero@uaslp.mx (M.d.l.L.G.-G.); catarina.loredo@uaslp.mx (C.L.-O.); 2 Soil and Water Laboratory, Faculty of Engineering, Universidad Autónoma de San Luis Potosí, San Luis Potosí CP 78290, SLP., Mexico; marcos.loredo@uaslp.mx; 3 Environmental Geochemistry Laboratory, Institute of Geology, Universidad Autónoma de San Luis Potosí, San Luis Potosí CP 78290, SLP., Mexico; maria.garcia@uaslp.mx; 4 Faculty of Chemical Sciences, Universidad Autónoma de San Luis Potosí, Soledad de Graciano Sánchez CP 78210, SLP., Mexico; erika.padilla@uaslp.mx

**Keywords:** abiotic stress, garlic, mercury soil contamination

## Abstract

Heavy metal contamination in soils is a growing concern due to anthropogenic activities, and *Allium sativum* (garlic) has shown tolerance to mercury pollution. We analyzed the physiological and molecular responses of garlic cloves exposed to HgCl_2_ at 0, 5000, 23,000, and 46,000 mg/kg for 2, 3, and 4 h. The germination percentage was lower than 46,000 mg/kg Hg for 4 h. We also analyzed the expression levels of *NAC* transcription factors and found that *AsNAC11* had higher expression at 46,000 mg/kg at 2 h; *AsNAC17* was underexpressed and the maximum was at 2 h at 23,000 mg/kg. *AsNAC20* had the highest expression (30 times more than the control) at 3 and 4 h with 23,000 mg/Kg. *AsNAC27* showed the highest expression at 3 h with 23,000 mg/kg. The tissues exhibited a maximum Hg bioconcentration factor of 0.037 at 23,000 mg/kg, indicating moderate mercury absorption. However, at a concentration of 46,000 mg/kg, the BCF decreased to 0.023. Our in-silico analysis revealed that the analyzed *AsNACs* are associated with various abiotic stress responses. This study provides valuable insights into genes that could be utilized for genetic improvement to enhance crop resistance to mercury soil contamination.

## 1. Introduction

Agriculture is currently facing significant challenges due to high concentrations of heavy metals (HMs) in soils, leading to soil acidification, reduced microbial communities, and plant toxicity [1]. The rise in HM levels is closely associated with anthropogenic activities [2], as elements such as lead (Pb), mercury (Hg), and cadmium (Cd) enter the environment through industrial waste [3]. Mercury, a heavy metal, is considered the third most toxic element on Earth [4]. According to the US-EPA [5], between 5000 and 8000 metric tons of mercury are released annually worldwide from various activities, including precious metal mining (38%), coal mining (24%), the production of non-ferrous materials (15%), and cement production (11%) with the remaining 12% being released as a result of different industries, such as metallurgy, paint production, and agrochemicals. In agricultural soils, the predominant form of mercury is its ionic state (Hg^2+^) [6]. Once released into the soil, mercury tends to remain in a solid state, binding to sulfides, clay particles, and organic matter. Studies have shown that Hg^2+^ can accumulate in both terrestrial and aquatic plants [7]. The primary effects of mercury toxicity in plants stem from its interaction with functional molecules; by disrupting the lipids in cell membranes, it alters their structure and function, affecting membrane fluidity and integrity, which impairs the regulation of substance transport [8]. Due to its high reactivity with sulfhydryl (thiol) groups, Hg exhibits a stronger binding affinity than Cd, As, and Pb, contributing to its heightened toxicity [9]. Additionally, mercury can replace essential ions such as Mg in chlorophyll [10], and its presence is strongly associated with oxidative stress in plant cells, leading to lipid peroxidation, protein and nucleic acid damage, and disruption of redox balance [11]. The addition of silicon to mercury-contaminated substrates can mitigate Hg absorption by reducing mercury availability in the medium while enhancing antioxidant defense mechanisms [12]. Mercury significantly inhibits seed germination in many plant species, though tolerance levels vary; for instance, in *Triticum aestivum* and *Cucumis sativus*, mercury caused the complete inhibition of germination at ≥1.5 mM in cucumber and at 1.7 mM in wheat [13]. Evaluating the effects of heavy metals at the germination stage is essential for understanding the impact on early plant development, with important implications for agriculture and environmental remediation.

The BCF (bioconcentration factor) is an index that quantifies the concentration of an element or compound in a plant relative to its concentration in the environment [14]. This index is essential for classifying plants as hyperaccumulators, accumulators, excluders, or indicators. Understanding a plant’s BCF provides insights into the transfer of contaminants from the environment to plants, with direct implications for public health, environmental protection, and the development of remediation strategies [15].

As sessile organisms, plants have developed various mechanisms to counteract the toxic effects of heavy metal (HM) exposure, with transcriptional regulation being a key factor in plant resistance to such stress. Currently, no studies have specifically explored the *NAC* (NAM, no apical meristem) gene from *Petunia hybrida*; the ATAF1/2 (transcriptional activator *1/2*) gene from *Arabidopsis thaliana*; and the CUC2 (cup-shaped cotyledon) gene from *Arabidopsis thaliana* in this context. In rice, *OsNAC2* and *OsNAC3* play critical roles in regulating seed germination through hormonal pathways such as ABA and ethylene, where *OsNAC2* delays germination while *OsNAC3* promotes it, presenting potential strategies for genetic improvement in crops. Similarly, in Arabidopsis, *ANAC092* influences germination under salt stress, further highlighting the importance of NAC factors in adaptation to environmental stress [16,17].

*NAC* transcription factors are present in a wide variety of plants and are involved in various functional processes, including responses to abiotic stress. These factors are characterized by a highly conserved *NAC* domain in their N-terminal region, which is essential for DNA binding. The *NAC* domain is divided into five subdomains (A, B, C, D, and E). Subdomains A, C, and D are the most conserved, with subdomain A being crucial for the formation of functional dimers, while C and D are responsible for DNA binding. Subdomains B and E are more variable, contributing to the functional diversity of these transcription factors [18,19]. In addition, the C-terminal region of *NAC* factors serves as a transcriptional regulatory region (TRR) with high variability, providing versatility in the regulation of gene expression.

Garlic (*Allium sativum*) is a significant group valued for its culinary and medicinal properties. Studies have shown that garlic is an effective chelating agent and can accumulate heavy metals (HMs) such as cadmium (Cd), lead (Pb), and mercury (Hg) in its tissues [20]. *Allium sativum* exhibits remarkable resistance to HM contamination through physiological and biochemical mechanisms that mitigate the damage caused by these pollutants. One of its most notable defense strategies is the ability to accumulate and detoxify HMs such as Cd, Pb, and Hg within its tissues, preventing these toxins from disrupting essential processes such as photosynthesis and cellular respiration. Garlic employs antioxidants, such as organosulfur compounds such as alliin, to neutralize reactive oxygen species (ROS) generated by HM-induced oxidative stress. In addition, *Allium sativum* produces phytochelatins and metallothioneins, proteins that chelate metal ions and facilitate their sequestration in vacuoles [21]. Notably, up to 95% of Pb and Cd accumulation in garlic occurs in the root, with smaller quantities in bulb and leaves. This accumulation pattern is closely linked to the exposure duration and the production of key enzymatic antioxidants, including superoxide dismutase (SOD), peroxidase (POD), and catalase (CAT), which play crucial roles in the plant’s defense against HM-induced stress [22,23].

This study aims to determine whether germination in garlic “seed cloves” is affected by high mercury concentrations in the growth medium and to investigate the involvement of selected *NAC* transcription factor genes from *Allium sativum* (*AsNAC11*, *AsNAC17*, *AsNAC20*, and *AsNAC27*) in stress response. We hypothesize that these NAC genes, previously identified by Wang et al. [24], exhibit a correlated response under mercury stress.

## 2. Materials and Methods

### 2.1. Plant Materials and Stress Treatments

Seed cloves of *Allium sativum* purple var., (Creole variety from SLP) sourced from the municipality of Ahualulco, San Luis Potosí, Mexico, were used in this study. Garlic cloves with similar weight and size characteristics were selected to ensure homogeneity. Seeds were sterilized with 10% NaClO for 10 min and then rinsed twice with sterile water for two minutes each time.

The mercury used in this study was in the form of mercury chloride (HgCl_2_). Four HgCl_2_ concentrations were selected for the experiments: 0, 5000, 23,000, and 46,000 mg/kg. These concentrations were chosen based on levels typically found in areas adjacent to mining waste. A control treatment with deionized water was also included. A previous experiment was conducted to observe germination percentages with prolonged mercury exposure times of 4, 8, 12, 16, and 24 h with the four concentrations mentioned. It was observed that only the 4 h exposure treatments showed germination; therefore, 4 h was considered the maximum exposure time for this study. The experimental design was completely randomized, consisting of 12 treatments. These treatments included three exposure times (2, 3, and 4 h), each combined with four different concentrations of mercury chloride (0, 5000, 23,000, and 46,000 mg/kg). Each treatment was conducted with three replicates, with each replicate containing 25 units, totaling 75 individuals per treatment. Seeds selected for the experiment were randomly assigned and immersed in the four HgCl_2_ concentrations for 2, 3, and 4 h. After exposure, the seeds were rinsed with deionized water. Ten samples were randomly selected for mercury concentration and gene expression analyses at time zero. The remaining seeds were planted in vitro on a water–agar substrate without any additional nutrients. Non-germinated containers were maintained in the absence of light at 27 °C.

### 2.2. Germination Percentage

Germination percentage was evaluated from 16 to 26 days after planting. The germination percentage for each treatment was calculated, considering *n* = 48 per treatment as 100%.

### 2.3. Analysis of AsNAC Genes

Four *AsNAC* genes were selected from the study previously conducted by Wang et al. [23] because they were the ones with the highest expression in the bulb under salinity stress conditions. Other reports have observed similar behaviors of these transcription factors under abiotic stress.

### 2.4. Phylogenetic Analysis and Classification of AsNAC Genes

To understand the phylogenetic relationship and to classify the *NAC* genes analyzed, the unrooted phylogenetic tree for the NAC proteins of *A. Sativum* (*AsNAC*) (4 genes) and *A. officinalis* (*AoNAC*) (85 genes) was constructed using MEGA 11 software (version 11.0.11). The *AsNAC* genes were classified according to their phylogenetic relationships with corresponding *A. officinalis* NAC members. All gene sequences were aligned using Muscle in MEGA 11 software (version 11.0.11), with the default parameters. The maximum likelihood (ML) method was used with the following parameters: 1000 iterations for the bootstrap method, the Poisson model, and all sites were used. Additionally, a phylogenetic tree of *AsNAC*, *AoNAC,* and their orthologs for *A. thaliana NAC*, as reported by Li et al. [25], was built with the same method. Gene sequences were obtained from TAIR [26], with the accession numbers reported by Ooka et al. [26], the *AoNAC* sequences from Li et al. [27], and *AsNAC* from Wang et al. [24].

### 2.5. Gene Structure and Motif Analysis of AsNAC Proteins

The online program MEME [28] was applied to analyze the conserved motifs in the AsNAC proteins with the following settings: maximum number of motifs—15, minimum motif width—6, maximum motif width—50, and number of repetitions—any.

### 2.6. CIS-Acting Elements on AsNAC Genes

To identify *cis*-acting regulatory elements, the promoter sequences of the *AsNAC* genes were analyzed using the PlantCARE [29] database. The identified elements were then organized graphically in Excel 16.92.

### 2.7. Protein Interactions

With the help of the freely available internet program STRING [30], the protein interactions of the three *AsNAC* genes with *A. thaliana* orthologs were established.

### 2.8. RNA Isolation from Allium sativum Seed Tissue

A total of 200 µL of TRIzol™ Reagent (ThermoFisher Scientific, Carlsbad, CA, USA; Cat. No. T-9424) was added to 4 mg of *A. sativum* seed tissue previously ground in liquid nitrogen, and total RNA extraction was carried out following the manufacturer’s instructions. The concentration and purity of the extracted RNA were measured using a NanoDrop One spectrophotometer. Subsequently, cDNA synthesis was performed.

### 2.9. cDNA Synthesis

Prior to cDNA synthesis, the RNA was treated with DNAse I (1 U/µL, RNase-Free; ThermoFisher Scientific; Cat. No. EN0521), following the manufacturer’s guidelines to eliminate any residual DNA. Subsequently, cDNA was generated from the purified and quantified RNA using the SuperScript™ IV Reverse Transcriptase Kit (ThermoFisher Scientific; Cat. No. 18091200), strictly adhering to the provided protocol. Oligo-random hexamer primers were utilized during this process. The synthesized cDNA was then stored at −70 °C until further use.

### 2.10. qPCR Conditions

The qPCR reactions were prepared in a total volume of 20 µL. The cDNA concentration was adjusted to 100 ng/µL. Each reaction included 1 µL of cDNA, 10 pmol of each primer, nuclease-free water, and 10 µL of the Maxima™ SYBR^®^ Green/ROX Kit mix (Thermo Scientific; Cat. No. K0222). Amplifications were performed on the QuantStudio 1 Real-Time PCR System (ThermoFisher Scientific) using the following thermal cycling conditions: an initial step of 2 min at 50 °C, 10 min at 95 °C, and then 40 cycles consisting of 15 s at 95 °C and 1 min at the annealing temperature specified in Table 1. Samples with cycle threshold (Ct) values above 39 were excluded from the analysis. Primer sequences were obtained from the study by Wang et al. [23], with detailed primer information listed in Table 1. Relative gene expression levels were calculated using the 2^−(∆∆Ct)^ method described by Livak and Schmittgen [31], using the *AsACTIN* gene as the normalization control.

### 2.11. Mercury Concentrations in Allium sativum Seed Tissue

Following the RT-qPCR analysis and interpretation of the results, we decided to measure the mercury concentration in samples from all HgCl_2_ treatments with a three-hour exposure period. Cold digestion was selected as the digestion technique due to its milder approach compared to other methods (e.g., hot digestion), minimizing the loss of volatile metals such as mercury. Samples were collected at two time points: immediately after exposure and five days post-exposure. A total of 0.5 g of tissue was weighed for each sample, with 10 biological replicates per treatment. The samples were ground and dehydrated at room temperature in a glass desiccator for four days. Subsequently, 2 mL of Suprapur-grade nitric acid (Merck, Darmstadt, Germany) was added, and the samples were placed in a water bath at 50 °C for 1 h, ensuring that the temperature did not exceed this limit. Samples were sonicated for 25 min and left to rest for 24 h. The digested samples were then diluted to 10 mL, and their weight was recorded (after pre-weighing the tubes and caps). Samples were stored at 2 °C until further analysis using inductively coupled plasma optical emission spectrometry (ICP-OES). The detection and quantification limits were experimentally determined using standards with concentrations starting at 0.05 μg L^−1^, following the *Eurachem Guide* [32]. The coefficient of determination (R^2^) was assessed using linear regression analysis based on calibration curve data, achieving an R^2^ value of ≥0.999. Analytical quality control was maintained by preparing certified reference materials, which were analyzed as control standards every 10 samples to evaluate instrument performance. The total mercury content per seed (TC) was calculated using the following equation:

TC = Hg concentration (mg Kg^−1^) × dry weight (g plant^−1^)

The limit of detection (LOD) and the limit of quantification (LOQ) are fundamental parameters to evaluate the performance of a method and ensure that the results obtained are reliable. For this research, formulas according to the *Eurachem Guide* [31] were used.

LOD = 3 × s’_0_

s’_0_ = Standard deviation obtained.

LOQ = kQ × s’_0_.

k: A statistical factor, usually 10.

s’_0_: Standard deviation of blank or low-concentration measurements.

In addition, we determined the BCF with the following formula:

BCF = (C_plant_) × (C_medium_)^−1^

C_plant_: Concentration in plant.

C_medium_: Concentration in medium.

### 2.12. Statistics

Data were analyzed using a two-way analysis of variance (ANOVA), followed by a Tukey *post hoc* test. All statistical analyses were performed using Statistica software, version 7.

## 3. Results

### 3.1. Germination Percentage

**Figure 1:**
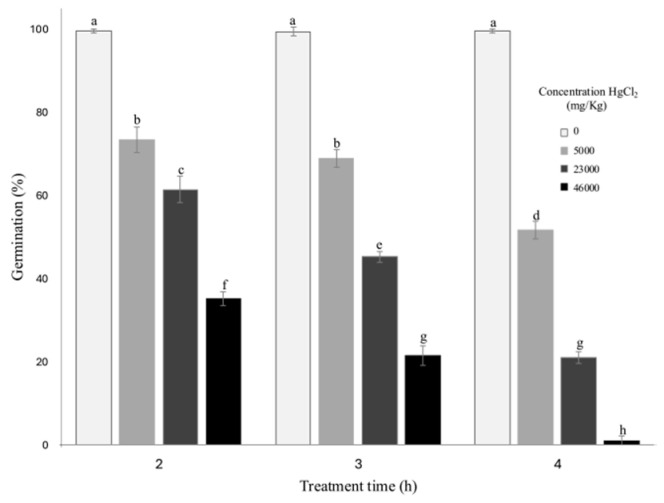
Germination analysis with three exposure times (2, 3, and 4 h) to mercury. Each bar is expressed as the means of 48 replicates ± SD. Different lowercase letters indicate significant differences at *p* < 0.05.

In the analysis of germination percentage, a strong negative correlation was observed across different treatments. Plants in the control group (0 mg/kg HgCl_2_) exhibited 100% germination (Figure 1), whereas the lowest germination rate (5%) was recorded at 46,000 mg/kg HgCl_2_ with 4 h of exposure. On the other hand, at the concentration of 5000 mg/kg HgCl_2_, no significant differences were observed between exposure times of 2 and 3 h; however, at 4 h, germination decreased significantly, even falling below that of the 23,000 mg/kg HgCl_2_ treatment with 2 h of exposure. This trend was consistent across different concentrations, suggesting that the exposure time has a greater impact on germination than the mercury concentration.

### 3.2. Phylogenetic Relationships and Classification of NAC Family TFs in Garlic

Due to the limited availability of species-specific information on garlic, we utilized well-studied species that share important key characteristics, especially related to their life cycle, environmental adaptation, and culinary and medicinal properties, to better understand gene function. To identify potential orthologs of the studied *NAC* genes, a phylogenetic analysis was performed. These shared traits reflect their classification within the order Asparagales and their ability to adapt and thrive in similar environmental conditions, as seen in asparagus (*Asparagus officinalis*).

**Figure 2:**
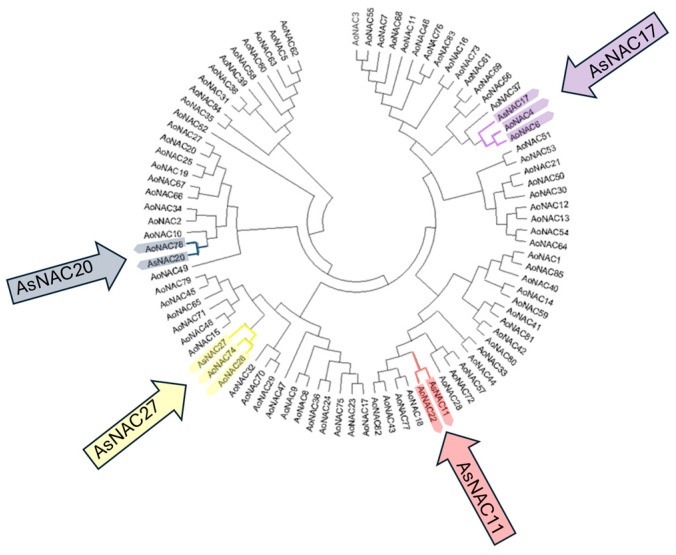
Phylogenetic tree of *NAC* genes between *A. officinalis* (*AoNAC*) and the four *AsNACs* analyzed. The *AsNAC* genes and their possible orthologs are indicated with colored arrows. The phylogenetic tree was compiled using the maximum likelihood (ML) method, with 1000 bootstrap replicates in the MEGA 11 program.

In the phylogenetic tree constructed in this study (Figure 2), which includes 85 previously reported genes [25] along with four selected *AsNACs*, six clades were formed, each containing one of the *AsNACs*. *AsNAC11* clustered closely with *AoNAC22* while *AsNAC17* grouped with *AoNAC4* and *AoNAC6*. *AsNAC20* was associated with *AoNAC78*, and *AsNAC27* clustered with *AoNAC74* and *AoNAC26*.

### 3.3. Conserved Motifs in NAC Genes

**Figure 3:**
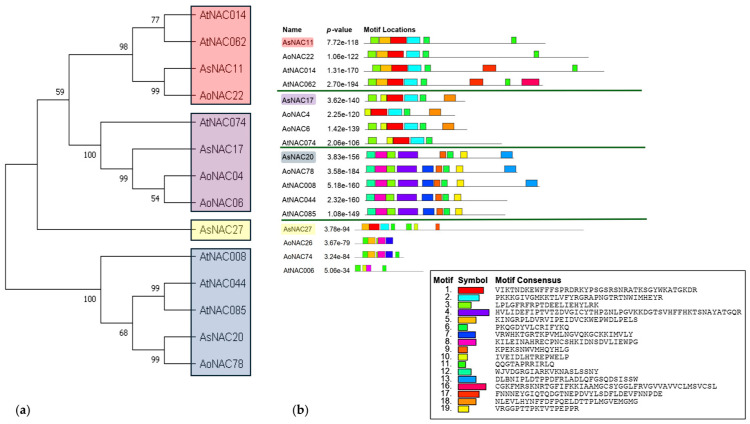
The conserved *AsNAC* sites evaluated in this work, as well as their potential orthologs in *Asparagus officinalis* (*AoNAC*) and *Arabidopsis thaliana* (*AtNAC*). (**a**) An unrooted phylogenetic tree was constructed using the ML method with 1000 bootstrap replicates based on *AsNAC*, *AoNAC*, and *AtNAC* gene-analyzed sequences. (**b**) The conserved AsNAC was analyzed, and potential orthologs protein motifs were predicted using the MEME program. Different colored boxes represent different motifs, and the black lines represent no conserved sequences. The scale bar is 200 amino acids.

To explore the structural diversity and similarities of *AsNAC* genes, conserved motifs were analyzed based on their phylogenetic relationships. A total of 19 conserved motifs were identified, numbered from 1 to 19 (Figure 3b). Genes in the same subgroup exhibited similar motif compositions, suggesting shared biological functions. Most *AsNAC* genes (Figure 3a) contained at least three well-conserved motifs at the N-terminal end associated with DNA binding. Notably, all analyzed AsNACs, except AsNAC27, possessed specific motifs at the C-terminal end, potentially conferring specialized functions.

### 3.4. CIS-Acting Elements on AsNAC Genes

*Cis*-acting elements are specific DNA regions within gene promoters that serve as binding sites for transcription factors. To elucidate the regulatory mechanisms of *AsNAC* genes in response to mercury-induced stress, we analyzed the transcriptional start site sequences of *AsNAC11*, *AsNAC17*, *AsNAC20*, and *AsNAC27*.

**Figure 4:**
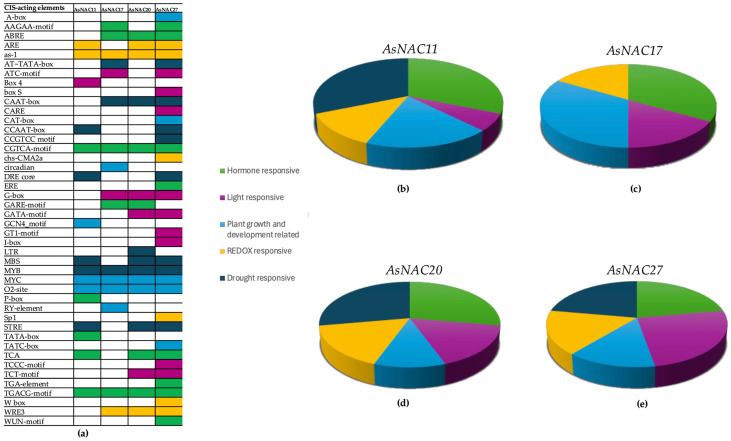
*Cis*-acting elements analyzed using the free online software *PlantCARE* (https://bioinformatics.psb.ugent.be/webtools/plantcare/html/, accessed on 5 June 2024). (**a**) Table showing the presence of *CIS*-acting elements in the promoter regions of the *AsNAC11*, *AsNAC17*, *AsNAC20*, and *AsNAC27* genes; (**b**) pie chart summarizing the distribution of *CIS*-acting elements categorized by their biological function in *AsNAC11*; (**c**) pie chart summarizing the distribution of *CIS*-acting elements categorized by their biological function in *AsNAC17*; (**d**) pie chart summarizing the distribution of *CIS*-acting elements categorized by their biological function in *AsNAC20*; and (**e**) pie chart summarizing the distribution of *CIS*-acting elements categorized by their biological function in *AsNAC27*.

Among the (*AsNAC11*: 16; *AsNAC17*: 16; *AsNAC20*: 18; and *AsNAC27*: 36) identified elements (Figure 4a), cis-acting elements were classified into five functional categories: (1) hormone response motifs (AAGAA motif, ABRE, CGTCA motif, ERE, GARE motif, P-box, TATA box, TCA, TGA element, TGACG motif, and WUN motif); (2) light response motifs (ATC-MOTIF, Box 4, Box S, CARE, G-box, GATA motif, GT1 motif, I-box, TCCC motif, and TCT motif); (3) plant growth and development motifs (A-box, CAT box, circadian, GCN4_motif, MYC, O_2_ site, RY element, and TATC box); (4) redox response motifs (ARE, as-1, chs-CMA2a, Sp1, W box, and WRE3); and (5) drought response motifs (AT~TATA box, CAAT box, CCAAT box, CCGTCC motif, DRE core, MBS, MYB, and STRE). To compare the numbers and types of cis-acting elements present in each A*sNAC* gene promoter, pie charts were generated (Figure 4b–d).

The analysis revealed that *AsNAC11* exhibits a high abundance of *cis*-acting elements, with a predominance of motifs related to hormone and drought responses, along with a significant number associated with plant growth and development. Conversely, *AsNAC17* shows a lower number of *cis*-acting elements compared to the other *AsNAC* genes, with a notable prevalence of motifs linked to plant growth and development.

*AsNAC20* exhibits a diverse distribution of cis-acting elements, similar to *AsNAC11*, with a relatively balanced representation of hormone response, light response, and plant growth and development motifs. Additionally, this gene is notable for the presence of motifs associated with redox response.

Finally, *AsNAC27* demonstrates a diversity in *cis*-acting element types comparable to *AsNAC20* and *AsNAC17*, with a higher representation of motifs associated with hormone and light responses. Additionally, it includes elements linked to drought response, suggesting a potential role in adaptation to abiotic stress conditions.

### 3.5. Analysis of the Interaction of AsNAC Proteins

**Figure 5:**
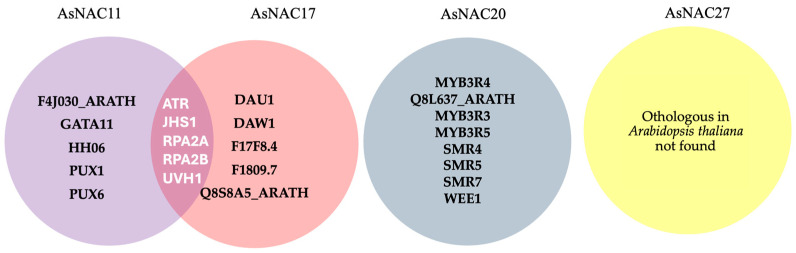
Venn diagram made with information from the freely available STRING database, using their ortholog proteins from *A. thaliana*. *AsNAC11* with their ortholog *AtNAC014*; *AsNAC17* with their ortholog *AtNAC074*, and *AsNAC20* with their ortholog *AtNAC044*. For *AsNAC27*, no ortholog genes were identified in *A. thaliana*.

This predictive analysis enabled the identification of proteins with the three *AsNAC* genes and analyzed their interactions. As shown in Figure 5, notable interactions were observed, with AsNAC17 and AsNAC11 exhibiting the highest number of connections. One key protein common to both is ATR, which, as highlighted in an a posteriori analysis (Table 2), plays a crucial role in cellular damage response. ATR is known to recognize the consensus sequence of the [ST]-Q substrate and phosphorylate the histone variant H2AX, forming H2AXS139ph at DNA damage sites, thereby regulating the DNA damage response mechanism [33].

Additionally, AsNAC11 and AsNAC17 share three other interactions with RPA2A, RPA2B, and UVH1, proteins involved in the repair of DNA damage caused by oxidative stress. However, for the F17F8.4, F1809.7, Q8S8A5_ARATH, and Q8S8A5_ARATH proteins, no reported function was identified.

In summary, the proteins interacting with the analyzed *AsNAC* genes primarily repair oxidative stress-induced DNA damage at various stages of the cell cycle. They also perform complementary functions, such as the removal of H_2_O_2_, aiding the cell in mitigating oxidative stress.

### 3.6. Expression Patterns of AsNAC Genes in Garlic Seed Cloves Exposed to High Mercury Concentrations

**Figure 6:**
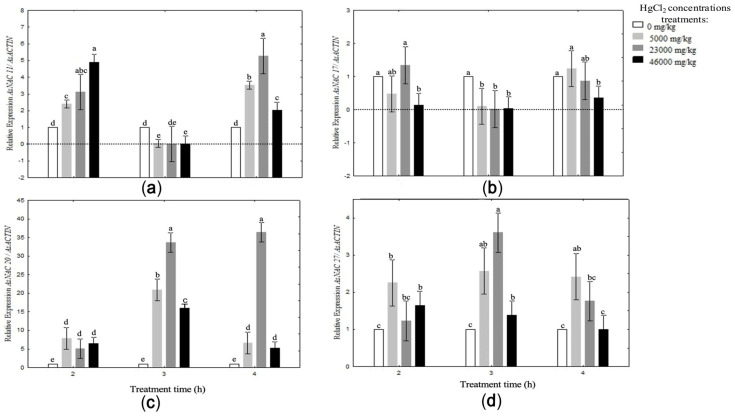
Expression patterns of *AsNAC* genes in garlic cloves under mercury stress. Each bar is expressed by the means of three replicates ± SD. Different lowercase letters indicate significant differences at *p* < 0.05. (**a**) Relative expression of *AsNAC11*/*AsACTIN*; (**b**) relative expression of *AsNAC17*/*AsACTIN*; (**c**) relative expression of *AsNAC20*/*AsACTIN*; and (**d**) relative expression of *AsNAC27*/*AsACTIN.*

Each analyzed *AsNAC* gene exhibited distinct expression patterns in response to mercury exposure (Figure 6), with *AsNAC20* and *AsNAC27* showing the most pronounced responses.

*AsNAC11* expression increased significantly with rising HgCl_2_ concentrations, peaking at 46,000 mg/kg after 2 h of exposure, followed by a slight decline at 3 h. At 4 h, its highest expression was observed at 23,000 mg/kg, surpassing the levels recorded at 46,000 mg/kg after 2 h.

*AsNAC17* showed a mild induction at the lowest mercury concentration (5000 mg/kg) but showed no significant upregulation at higher concentrations. Its expression pattern varied according to concentration and exposure time; at 23,000 mg/kg and 2 h, expression was statistically similar to the control. However, at other concentrations and time points (3 and 4 h), the gene was downregulated. This suggests that *AsNAC17* plays a role in early, transient responses to mercury stress, potentially participating in the activation of stress perception pathways, with expression decreasing over time.

The general expression trend for *AsNAC11*, *AsNAC17*, and *AsNAC27* across the tested concentrations was characterized by an initial increase at 2 h, a decrease at 3 h, and a subsequent increase at 4 h. In contrast, *AsNAC20* displayed a unique pattern, showing a robust and marked induction, particularly at 23,000 mg/kg after 3 h of mercury exposure. This gene demonstrated the strongest upregulation among all the *AsNAC* genes analyzed.

Similarly, *AsNAC27* was strongly induced at higher HgCl_2_ concentrations, peaking at 3 h. Although its expression at lower concentrations was less intense, it remained significantly higher than the control group.

These results emphasize the distinct roles of *AsNAC* genes in response to mercury stress, with *AsNAC20* and *AsNAC27* likely serving as key regulators of defense mechanisms under severe stress conditions.

### 3.7. Mercury Concentrations in Garlic Seed Clove Tissue

**Figure 7:**
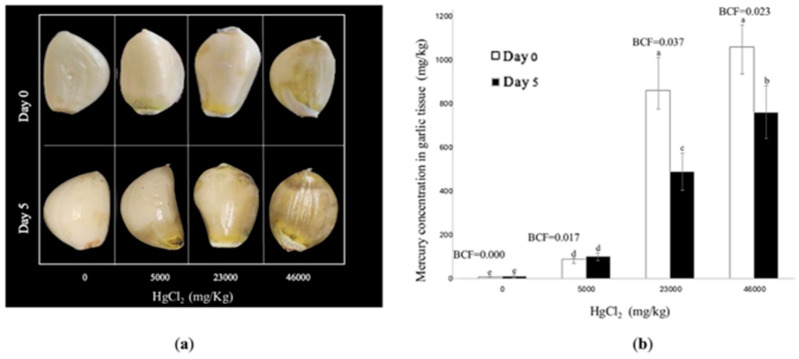
Mercury concentration in garlic tissue. (**a**) Images of garlic seeds exposed to four different concentrations of HgCl2 (0, 5000, 23,000, and 46,000 mg/kg), showing the effect of mercury on the tissue at 0 and 5 days. (**b**) Mercury concentrations in garlic seed tissue were analyzed using ICP-optical at 0 and 5 days. Each bar is expressed by the means of ten replicates ± SD. Different lowercase letters indicate significant differences at *p* < 0.05. Bioconcentration factor (BCF) values were also calculated for each treatment at 0 days.

Mercury accumulation in garlic clove tissue increased proportionally with higher HgCl_2_ concentrations (Figure 7b). At the highest concentration (46,000 mg/kg HgCl_2_), mercury levels in the tissue were significantly elevated at both 0 and 5 days.

The bioconcentration factor (BCF) peaked at an intermediate concentration (23,000 mg/kg), reaching a maximum value of 0.037, indicating moderate mercury uptake efficiency. However, at the highest concentration (46,000 mg/kg), the BCF slightly decreased to 0.023, suggesting the activation of saturation or toxicity mechanisms that may limit further absorption. The limit of detection (LOD) was determined to be 0.33 mg/kg, while the limit of quantification (LOQ) was 1 mg/kg.

Visual assessments of garlic cloves under different HgCl_2_ concentrations (Figure 7a) revealed that, in the control group (0 mg/kg), garlic cloves remained in good condition, with no visible damage or significant changes in appearance. On day 0, garlic cloves exposed to higher HgCl_2_ concentrations appeared almost normal, even at elevated levels. However, at 5 days, cloves treated with the highest concentrations (23,000 and 46,000 mg/kg) exhibited clear signs of damage, including discoloration, yellowish patches, and necrotic areas. These results highlight the concentration-dependent effects of HgCl_2_ on garlic tissue, with visual damage becoming evident with prolonged exposure to high mercury levels.

## 4. Discussion

In this study, *Allium sativum* demonstrated remarkable resilience to mercury (Hg), an extremely toxic heavy metal (HM) known to disrupt numerous plant physiological processes, including seed germination. Despite the high toxicity of mercury, *A. sativum* showed resistance to elevated concentrations. As illustrated in Figure 1, germination rates remained above 60% at mercury concentrations up to 23,000 mg/kg. However, as Hg concentrations increased, the germination percentage progressively declined, becoming as low as 5% at 46,000 mg/kg. This decline highlights the toxic effects of mercury on seed viability. While the precise mechanisms disrupted by mercury during seed germination and embryo development remain unclear, it is known that mercury replaces -SH groups in proteins, interfering with essential enzymes such as amylase and protease, which are both critical for germination [9]. Furthermore, mercury induces cellular water deficits in seed cells, leading to the inhibition of root and shoot growth [46]. This study underscores the unique capacity of *A. sativum* to endure high mercury concentrations, while also shedding light on the detrimental effects of this HM on critical physiological processes during germination.

HM exposure triggers complex transcriptional regulatory mechanisms in plants. In this study, the in silico and transcriptional expression analyses of *NAC* genes provided insights into their function in *Allium sativum* under mercury-induced stress.

Protein interaction analysis (Table 2) revealed that *AsNAC11* plays a significant role in repairing DNA damage caused by oxidative stress. Additionally, cis-acting elements associated with plant growth, development, and hormone signaling were identified, including abscisic acid response elements (ABREs), which are well-documented in *Oryza sativa* as regulators of stress-induced genes, particularly those involved in cellular protection under conditions of drought, cold, and salinity [47]. Additionally, the presence of the TCA element, a binding site for salicylate-induced transcription factors reported in *Vitis vinifera* [48], suggests that AsNAC11 is mediated by hormonal signaling pathways involving the salicylic acid (SA) pathway. SA is critical for activating plant defense responses, particularly through the production of reactive oxygen species (ROS) and the induction of antioxidant responses, which are essential to counteract oxidative stress caused by mercury. Our results showed that the expression levels of *AsNAC11* were significantly elevated, up to five times higher than the control, under treatments with 46,000 mg/kg HgCl_2_ for 2 h and 23,000 mg/kg for 4 h. Phylogenetic analysis revealed that *AsNAC11* is an ortholog of *AtNAC056*, a gene previously identified as a key regulator of oxidative stress responses and senescence induction in *Arabidopsis thaliana* under drought conditions [49]. These findings suggest that *AsNAC11* may play a role in regulating defense mechanisms against oxidative stress and modulating cellular aging processes.

*AsNAC17* was found to interact with multiple proteins (Table 2) involved in DNA damage repair and abiotic stress defense (drought and salinity), including ATR, RPA2A, RPA2B, and UVH1. These proteins are also implicated in gibberellin (GA) signaling (PUX1 and PUX6) and jasmonic acid (JA) pathways, as well as transcription factor (TF) regulation, in *Capsicum annuum*. Additionally, *AsNAC17* interacts with F4J030_ARATH, a heat shock protein (HSP) [50] that has been studied in *Zea mays* and is known to protect the electron transport chain in the photosynthetic system under thermal, oxidative, and light stress. HSPs have also been reported to mitigate heavy-metal-induced stress, including lead (Pb) and nickel (Ni) exposure [51].

Light is not only essential for photosynthesis but also plays a role in stress regulation. The cis-acting element analysis showed that *AsNAC17* participates in light-responsive pathways. Regulatory elements such as G-box and GT1, which are critical for light-dependent gene regulation in *Nicotiana tabacum* [51], were identified. Given that mercury disrupts photosynthesis by replacing the Mg ion in the pyrrole rings of chlorophyll, these light-dependent elements may help modulate *AsNAC* gene expression to minimize damage to light-harvesting complexes (LHCs) [13]. In *Arabidopsis thaliana*, the G-box has been identified as a binding site for transcription factors that regulate antioxidant synthesis, which are essential for mitigating oxidative stress caused by mercury [51].

Phylogenetic analysis identified *AsNAC17* as an ortholog of *AtNAC017* in *A. thaliana*. *AtNAC017* regulates oxidative stress signaling through a retrograde pathway from mitochondria to the nucleus, activating genes that protect cells against oxidative damage [52,53]. In our relative expression analysis, *AsNAC17* showed downregulation in all mercury treatments, except at 23,000 mg/kg for 2 h and 5000 mg/kg for 4 h. This suggests that *AsNAC17* may act in the early stages of the mercury stress response, activating signaling pathways that limit cellular damage caused by oxidative stress.

Among the analyzed NAC genes, *AsNAC20* was identified as one of the most intriguing transcripts in garlic’s response to mercury stress, displaying high expression levels, with a maximum induction of 38-fold compared to the control under the 23,000 mg/kg HgCl_2_ treatment at 3 and 4 h of exposure. Unlike the other three *AsNACs,* most of the cis-acting elements in *AsNAC20* are not related to growth and development. Instead, hormone- and light-responsive elements predominate, suggesting that this gene is tailored to counteract the effects of oxidative stress. Phylogenetic analysis linked *AsNAC20* to *AtNAC072* (RD26), a transcription factor known to respond to dehydration, salinity, and drought [54]. Additionally, the proteins associated with *AsNAC20* (Table 2) are primarily involved in cell cycle regulation in response to DNA damage and NAD(P)H regulation under various abiotic stress conditions, including salinity and drought. These results suggest that *AsNAC20* may play a role in detoxification processes and the regulation of cellular water balance under heavy metal stress conditions.

The phylogenetic analysis of the *AsNAC27* gene yielded inconclusive results. In the subsequent MEME analysis of conserved motif structures, the genes *AoNAC74* and *AoNAC26*, identified as phylogenetic relatives, did not exhibit a similar structural configuration to *AsNAC27*. Wang et al. [24] described the *AsNAC* gene family, reporting 46 members. In their characterization efforts, *AsNAC27* was classified as an outlier due to its unique structural features. Cis-acting element analysis revealed that *AsNAC27* has the highest number of elements among the four analyzed genes, with a total of 48. The primary function of these elements relates to growth and development, followed by light response, with a minor proportion (5%) associated with anaerobic induction. Relative expression levels of *AsNAC27* were up to four times higher than the control under the 23,000 mg/kg HgCl_2_ treatment after 3 h of exposure.

Our results from the mercury concentration analysis in *A. sativum* tissue indicated that mercury accumulation depends on its concentration in the medium. Garlic bulbs were able to reduce mercury levels within their tissue by up to 40% (under the 23,000 mg/kg HgCl_2_ treatment) from day zero to day five. At 5000 mg/kg HgCl_2_, the seeds exhibited a low capacity for mercury accumulation, a behavior consistent with previous studies suggesting that plants may develop physiological barriers to heavy metal absorption at relatively low concentrations. In a previous study on *A. sativum*, Carabulea et al. [55] demonstrated significant correlations between soil concentrations of Cd, Zn, and Pb and their accumulation in garlic bulbs, with *r* values of 0.775, 0.649, and 0.423, respectively, while the correlation for Cu was not significant (*r* = 0.274). This indicates that HM accumulation is differential. Similarly, Tegegne and Mengiste [56] measured concentrations of Cd, Zn, Pb, and Cu in *A. sativum* and observed higher metal accumulation in stem and leaf tissues than in bulbs for all examined metals.

Although specific mechanisms for HM tolerance in *A. sativum* are not well-documented, some mechanisms have been characterized for lead (Pb). These include the immobilization of lead ions in cell walls, synthesis of cysteine-rich proteins, significant vacuolization in the cytoplasm, and formation of dense granules in vacuoles and mitochondrial membranes. This suggests that vacuoles are the primary organelles for lead storage in *A. sativum* [57].

Finally, the bioconcentration factor (BCF) analysis indicated that *A. sativum* seeds have a limited capacity for mercury accumulation. The BCF values obtained were 0.017, 0.037, and 0.023 for 5000, 23,000, and 46,000 mg/kg HgCl_2_, respectively, suggesting a moderate ability to uptake mercury.

## 5. Conclusions

In this study, *Allium sativum* demonstrated resilience to mercury (Hg) toxicity, an extremely harmful heavy metal known to disrupt various plant physiological processes such as seed germination. These findings suggest potential as a promising species for cultivation in soils contaminated with HM. However, the limited bioconcentration capacity observed in this study indicates that garlic does not function as a strong mercury accumulator, which could reduce its effectiveness for phytoremediation in highly contaminated environments.

The identification and characterization of cis-regulatory elements in AsNAC gene promoters provide new insights into plant modulation and their responses to abiotic stress, including HM toxicity. This study also suggests that *NAC* transcription factors play a key role in regulating mercury stress responses by integrating hormonal, light, and developmental signals to optimize plant survival. The phylogenetic similarity of *AsNAC* genes to orthologs in other species highlights the possible conservation of stress-regulatory functions across different plants, opening new research avenues for the genetic improvement of crops grown in contaminated soils.

Finally, further investigation into *Allium sativum* during other phenological stages is needed to better understand its resistance and accumulation capacities for HMs, as well as its potential for the phytoremediation of contaminated soils. Understanding these mechanisms paves the way for novel genetic improvement strategies that could enhance crop resilience to the growing threat of HM contamination in agricultural ecosystems.

## Figures and Tables

**Table 1:** Primer sequences used for qRT-PCR and their alignment temperatures (°C).

Gene	Forward Primer (5′–3′)	Reverse Primer (5′–3′)	Alignment Temp (°C)
*AsNAC11*	CTACACCATTGAACCAAGCATCTCC	GAGCACTTCATCATTAGCCACATTACA	59
*AsNAC17*	CTCATACACCACCTAAGGAGGACTG	CCGAAGCATCCACCTAACATTGATTG	59
*AsNAC20*	CAAGAGAAGAAGAGATGGAGCAAGTCA	CAACTAGATATGCTGTCCTGAGAACCA	61
*AsNAC27*	GCTTGGTACACTGCAACGGTAGTAA	TTGACTTCTCGGACTGGAGGATGG	61
*AsACTIN*	TGCTCTGGATTATGAACAGGAACTTGA	CAATCATTGAAGGCTGGAACAACACT	58

**Table 2:** Descriptions of the functions of the proteins related to the three different AsNAC proteins analyzed.

AsNAC	Protein	Function	References
11	DAU1	Key role in regulating sperm cell development.	[30]
11	DAW1	Preserved in plants and involved in the regulation of cell polarity and growth.	[34]
11, 17	JHS1	This gene plays a crucial role in the response to DNA damage, specifically in the repair of double-strand breaks, and helps maintain the integrity of the root and shoot apical meristem (RAM and SAM).	[35]
11, 17	ATR	Plays a central role in cell cycle regulation by transmitting DNA damage signals to downstream effectors of cell cycle progression.	[33]
11, 17	RPA2A, RPA2B	Involved in the repair of DNA lesions, particularly those resulting from oxidative stress.	[36]
11, 17	UVH1	Involved in nucleotide excision repair (NER) of damaged DNA (dark repair mechanism). Involved in the repair of UV light and probably oxidative damage.	[37]
17	F4J030_ARATH	Member of the small heat shock proteins (sHSPs). In addition to heat stress, sHSPs can also be induced by other types of abiotic stress, such as dehydration, salinity, and oxidative stress.	[38]
17	GATA11	The main function of GATA TFs is to regulate gene expression in response to environmental and hormonal stimuli, as well as in developmental processes.	[39]
17	HH06	It associates with basic helix–loop–helix (bHLH) transcription factors, allowing the formation of dimers that regulate genes involved in hormonal signaling and in response to abiotic stresses through the synthesis of anthocyanins.	[40]
17	PUX1, PUX6	These play crucial roles in regulating the structure and function of essential proteins such as CDC48 and in modulating GA hormone signaling.	[41]
20	MYB3R3, MYB3R4, MYB3R5	The MYB family is involved in diverse processes such as developmental control, the determination of cell fate, plant responses to environmental factors and hormones, signal transduction in plant growth processes, pathogen defense, and xylogenesis and lignin biosynthesis.	[42]
20	Q8L637_ARATH	A pyridoxamine 5′-phosphate oxidase family protein involved in the regulation of cellular metabolism. They can oxidize 6-NADH and 6-NADPH, suggesting a role in the elimination of damaged forms of NAD(P)H.	[43]
20	SMR5, SMR7	These SIAMESE-RELATION (SMR)-type regulators modulate cell cycle arrest in response to DNA damage or oxidative stress. SMR5 has been shown to play a crucial role in cell cycle arrest in situations of water or genotoxic stress.	[44]
20	WEE1	Encodes a protein kinase that plays a crucial role in regulating the cell cycle.	[45]

## Data Availability

The original contributions presented in this study are included in the article. Further inquiries can be directed to the corresponding author.
